# Artificial Intelligence Systems for Diagnosis and Clinical Classification of COVID-19

**DOI:** 10.3389/fmicb.2021.729455

**Published:** 2021-09-27

**Authors:** Lan Yu, Xiaoli Shi, Xiaoling Liu, Wen Jin, Xiaoqing Jia, Shuxue Xi, Ailan Wang, Tianbao Li, Xiao Zhang, Geng Tian, Dejun Sun

**Affiliations:** ^1^Clinical Medical Research Center/Inner Mongolia Key Laboratory of Gene Regulation of the Metabolic Diseases, Inner Mongolia People’s Hospital, Hohhot, China; ^2^Department of Endocrinology, Inner Mongolia People’s Hospital, Hohhot, China; ^3^Geneis (Beijing) Co., Ltd., Beijing, China; ^4^Qingdao Geneis Institute of Big Data Mining and Precision Medicine, Qingdao, China; ^5^Department of Otolaryngology, Inner Mongolia People’s Hospital, Hohhot, China; ^6^Baotou City Hospital for Infectious Diseases, Baotou, China; ^7^Department of Pulmonary and Critical Care Medicine/Key Laboratory of National Health Commission for the Diagnosis & Treatment of COPD, Inner Mongolia People’s Hospital, Hohhot, China

**Keywords:** COVID-19, CT images, laboratory findings, artificial intelligence, correlation analysis

## Abstract

**Objectives:** COVID-19 is highly infectious and has been widely spread worldwide, with more than 159 million confirmed cases and more than 3 million deaths as of May 11, 2021. It has become a serious public health event threatening people’s lives and safety. Due to the rapid transmission and long incubation period, shortage of medical resources would easily occur in the short term of discovering disease cases. Therefore, we aimed to construct an artificial intelligent framework to rapidly distinguish patients with COVID-19 from common pneumonia and non-pneumonia populations based on computed tomography (CT) images. Furthermore, we explored artificial intelligence (AI) algorithms to integrate CT features and laboratory findings on admission to predict the clinical classification of COVID-19. This will ease the burden of doctors in this emergency period and aid them to perform timely and appropriate treatment on patients.

**Methods:** We collected all CT images and clinical data of novel coronavirus pneumonia cases in Inner Mongolia, including domestic cases and those imported from abroad; then, three models based on transfer learning to distinguish COVID-19 from other pneumonia and non-pneumonia population were developed. In addition, CT features and laboratory findings on admission were combined to predict clinical types of COVID-19 using AI algorithms. Lastly, Spearman’s correlation test was applied to study correlations of CT characteristics and laboratory findings.

**Results:** Among three models to distinguish COVID-19 based on CT, vgg19 showed excellent diagnostic performance, with area under the curve (AUC) of the receiver operating characteristic (ROC) curve at 95%. Together with laboratory findings, we were able to predict clinical types of COVID-19 with AUC of the ROC curve at 90%. Furthermore, biochemical markers, such as C-reactive protein (CRP), LYM, and lactic dehydrogenase (LDH) were identified and correlated with CT features.

**Conclusion:** We developed an AI model to identify patients who were positive for COVID-19 according to the results of the first CT examination after admission and predict the progression combined with laboratory findings. In addition, we obtained important clinical characteristics that correlated with the CT image features. Together, our AI system could rapidly diagnose COVID-19 and predict clinical types to assist clinicians perform appropriate clinical management.

## Introduction

In December 2019, a cluster of patients with unidentified pneumonia disease was discovered. Soon a novel coronavirus was isolated from these patients, which belonged to the beta-coronavirus family and was named severe acute respiratory syndrome coronavirus 2 (SARS-CoV-2) (Zhu et al., 2020). On February 12, 2020, the World Health Organization (WHO) named the disease as Coronavirus Disease 2019 (COVID-19) as it had spread quickly all over the world and developed into a plague (Mahase, 2020). By May 11, 2021, more than 159 million people were confirmed infected with more than 3 million cases of mortality. The crowd was affected easily, and the common clinical manifestations were fever, dry cough, and fatigue (Chen et al., 2020; Huang et al., 2020). Mild patients may have no obvious clinical symptoms, while severe patients may have dyspnea and hypoxemia. The incubation period of COVID-19 was 1–14 days, mostly 3–7 days, and it was infectious even in the incubation period (Sun et al., 2020). The main transmission route was *via* respiratory droplets and close contact (Li et al., 2021). Patients with COVID-19 but with no symptoms may have transmitted the virus to close contacts before a definite diagnosis could be made. Though there are many studies on identifying effective drugs against SARS-CoV-2 (Tang et al., 2020; Peng et al., 2021), most of them need further experimental and clinical validation (Peng et al., 2020; Zhou et al., 2020). Therefore, early diagnosis was extremely important.

According to the current diagnostic criteria, the gold standard of diagnosis was nucleic acid detection, and reverse transcription–polymerase chain reaction (RT-PCR) had become the main test method because of its low cost and high speed compared to complete genome sequencing (Loeffelholz and Tang, 2020). However, due to sampling deviation, with low viral load (the number of virus replications cannot reach the qPCR detection threshold) in the specimen and the accuracy of detection reagents, the detection results may be false negative (Ai et al., 2020; Mei et al., 2020; Rubin et al., 2020), which resulted in suspected patients not being identified and isolated in time. This would lead to further spread of infection, resulting in the epidemic being difficult to control. On the other hand, patients with COVID-19 could not get timely treatment if not identified, so a series of nucleic acid tests may be required to eliminate the possibility of false-negative results in suspected or discharged patients. During the outbreak of a highly infectious epidemic, a novel method for rapid and accurate diagnosis of patients with COVID-19 was urgently needed.

Chest computed tomography (CT) examination was an indispensable method in the diagnosis of COVID-19, and CT could reveal the severity of COVID-19 as well as the disease progression in dynamic monitoring, while nucleic acid detection was only a qualitative test result (Dai et al., 2020). In the critical epidemic situation of Wuhan, China, CT played an important role for patients who had negative nucleic acid tests but with symptoms or close contact with the confirmed patients. The most common CT findings in patients affected by COVID-19 included ground glass opacities (GGO) and consolidation involving the bilateral lungs in a peripheral distribution (Zhang et al., 2020). Pleural effusions, lymphadenopathy, and discrete pulmonary nodules were very rare (Kanne, 2020; Nishiura et al., 2020; Song et al., 2020). Consolidation was considered as a sign of disease progression. However, CT alone was not suitable for independently ruling out SARS-CoV-2 infection to the best of our knowledge because some patients may have normal radiological features at early stages of the disease and doctors could not distinguish by naked eye observation (Chung et al., 2020). During the period of the epidemic, the physicians needed to analyze numerous CT images to judge the condition of patients and integrated them with clinical information to make the final judgment. Thus, developing artificial intelligence (AI)-based imaging analysis methods was crucial to support physicians. Mei et al. (2020) had established an AI algorithm to combine chest CT findings with clinical symptoms, laboratory testing, and exposure history to rapidly diagnose patients with SARS-CoV-2.

In this study, we used AI algorithms to construct a model to attempt to distinguish patients with COVID-19 from common pneumonia and non-pneumonia based on CT images rapidly and accurately, which would greatly reduce the workload of radiologists and also brought huge convenience to the hospitals without experienced radiologists. It was convenient for doctors to take timely and accurate pertinent treatment and improve their prognosis. Furthermore, we could predict the progression of COVID-19 combined with laboratory findings on admission, and this would be very meaningful for medical workers to take appropriate treatment.

## Materials and Methods

### Data Collection

This retrospective study had been approved by the ethics committees of the Inner Mongolia People’s Hospital. Further informed consent was waived with approval, as the study only involved de-identified data and had no potential risk to patients.

We collected chest CT images and clinical information from all confirmed COVID-19 patients who were admitted to different hospitals in Inner Mongolia. All patients had been confirmed COVID-19 positive by nucleic acid detection. In addition, novel coronavirus pneumonia cases that entered Inner Mongolia from overseas were also included in the study. The demographic characteristics, clinical features of first detection, and initial chest CT images were sorted out. Other pneumonia (not COVID-19) and non-pneumonia patients were randomly selected from hospitals within the last 6 months before COVID-19 occurred.

### Preprocessing and Image Augmentation

Qualified CT slices were selected by senior radiologists from the original hundreds of images produced by CT scanners. Pulmonary tissue <20% of the size of the body part and the images containing severe artifacts or obvious image resolution reductions were excluded. Finally, we selected 1,041 chest CT images from 150 non-pneumonia patients, 965 CT images from 186 patients with COVID-19, including 61 mild and 125 moderate types, and 852 CT images from 113 other pneumonia patients. The raw CT images with 512 × 512 pixels were rescaled to the size of 224 × 224 pixels, after which we normalized image channels, respectively.

To alleviate an overfitting phenomenon, data augmentation is employed. By applying horizontal flipping image data, we doubled the number of images in the training data set.

### Model Construction Using Transfer Learning

To build an automated system with two progressive models, the first model is used for diagnosing COVID-19, and the second is for disease typing. For the first model, a one-vs.-the-rest classification strategy is employed; more specifically, the CT data were divided into one (COVID-19) vs. other two classes that include other pneumonia cases and normal control cases. Fairly, fivefold cross-validation is used for performance evaluation (Kaczorowska et al., 2021). Patients with each disease type were divided into five subgroups with stratified sampling. In our experimental setup, we loaded three pre-trained models (resnet18, vgg19, and vgg16) on the ImageNet database and reset the size of the final fully connected layer. Training was terminated when the validation accuracy did not increase for 10 epochs. Transfer learning was a popular method in computer vision community since it enabled an accurate model to be established in a short time (Rawat and Wang, 2017).

For the model combining CT images and laboratory findings, we applied the global averaging layer to the last layers of the convolutional model described previously to derive a 512 dimensional feature vector for representation. The way we used in multimodal fusion is Compact Bilinear Pooling (CBP). A total of 55 laboratory findings of the same patient were concatenated with this feature vector. A Multi-Layer Perceptron (MLP) took this combined feature vector as the input to predict the status of COVID-19. We used a three-layer MLP; each layer has 64 nodes followed by a batch normalization layer, a fully connected layer, and a ReLU activation function. The MLP was trained with an end-to-end manner. Then, we applied binary cross-entropy loss function to evaluate both MLP and CNN during the feed forward phase. Eventually, we evaluated the performance of models based on four metrics, which include area under the curve (AUC) of receiver operating characteristic (ROC), sensitivity, specificity, and accuracy.

### Correlation Analysis Between Imaging Features and Laboratory Findings

Clinical records collected from patients with COVID-19 included demographic characteristics, such as gender and age, clinical data on vital signs and symptoms, as well as dynamic results of laboratory test and imaging data monitored from admission to discharge. Laboratory tests included routine blood tests, liver and kidney biochemical indicators, coagulation function tests, and serum protein levels and activities.

To explore the correlation between lung CT image features and laboratory detection indexes, Spearman’s correlation test was applied. A total of 512 CT features and 55 experimental detection indicators were input. The resultant correlation was considered significant when *p* < 0.05 after correction with the Holm–Bonferroni method.

## Results

### Imaging Protocol

**FIGURE 1:**
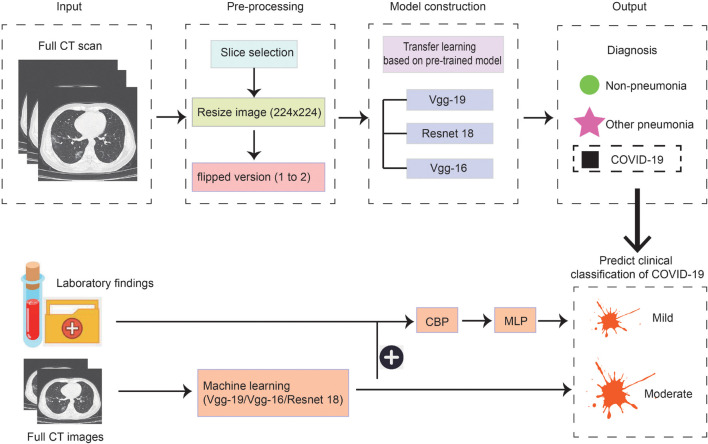
Scheme of our deep learning framework based on the first chest CT images and laboratory findings for the diagnosis and classification of COVID-19. The first row describes the process of constructing the diagnosis model. Full CT images of COVID-19, other pneumonia, and non-pneumonia were taken as input and generated the probability of three disease states with classification networks. The second row indicates predicting the clinical classification of COVID-19 based on CT images only and based on CT images together with laboratory findings.

As the methods used in model construction were relatively complex and multiple threads were covered in this study, we drew a flowchart to illustrate the experimental process (Figure 1). Firstly, we used three kinds of methods to construct models to distinguish patients with COVID-19, other common pneumonia, and normal controls based on the chest CT scans, then the model was built to further classify the types of COVID-19 based on CT images on admission. Lastly, the laboratory findings were combined with the CT scans to improve the performance of machine-learning models to classify the types of COVID-19.

### Image Datasets of Patients

We collected all CT images of 237 patients with COVID-19 from admission to discharge in Inner Mongolia, with 79 domestic cases and 158 imported cases. Among them, seven patients belong to severe novel coronavirus pneumonia, 160 patients were moderate type, and 70 patients were mild type. We finally got 2,858 qualified CT slices taken into model construction after a selection performed by experienced radiologists, which were from 186 patients infected with SARS-CoV-2, 113 other pneumonia patients, and 150 normal control patients (Table 1). The severe type of COVID-19 was excluded, as the number of this kind of patients was too low. In addition, the number of mild type was significantly less than the moderate type. In order to get better training effect, we should make the number of CT films as close as possible.

**TABLE 1:** Summary of the patients’ information.

	Non-pneumonia	Other common pneumonia	COVID-19	
			Mild	Moderate
Patients	150	113	61	125
CT slices	1,041	852	495	470

### Results of the Diagnostic Model

**FIGURE 2:**
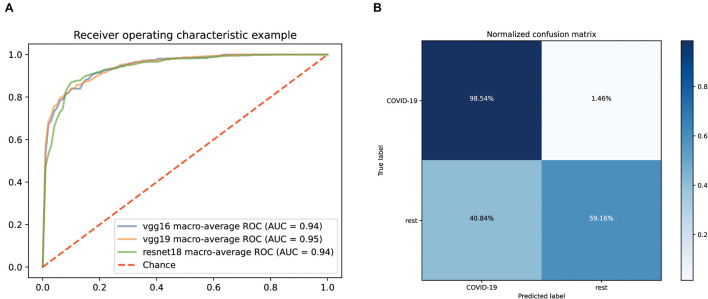
Performance of our AI systems in differentiating COVID-19 from other common pneumonia and normal controls. **(A)** ROC curves of vgg16, vgg19 and resnet18 systems. **(B)** Normalized confusion matrix of vgg19 system.

Normalized CT images were put into the model construction without the preprocessing of lung segmentation or feature selection. To distinguish COVID-19 from other common pneumonia and normal controls, we applied vgg19, resnet18, and vgg16 as a backbone to train the deep learning model, and the performance of the ROC curve of these AI models based on test data is demonstrated in Figure 2A. All models have achieved superior performance with AUC value of ROC curves between 0.94 and 0.95, and the AUC value of vgg19 presenting the highest. In addition, we calculated and compared the sensitivity, specificity, and accuracy of the three AI models. Among them, the best model vgg19 was able to distinguish COVID-19 from the other two classes with 78.85% accuracy, 98.54% sensitivity, and 59.16% specificity (Figure 2B).

**FIGURE 3:**
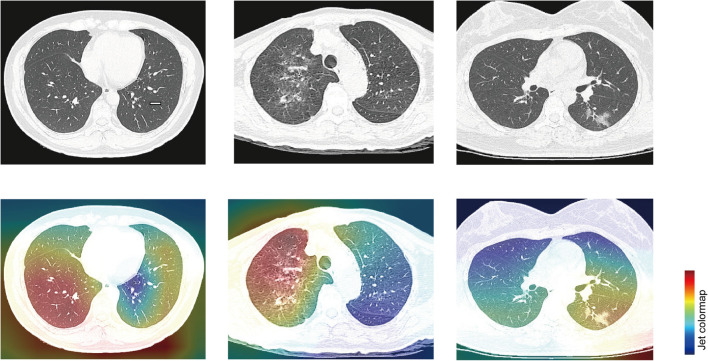
Typical examples of chest CT images of patients and visual features correlated to classification. The first row demonstrates the original images of patients with normal control, other pneumonia, and COVID-19, respectively. The second row demonstrates heat maps corresponding to the first row, showing highly relevant areas of classification.

In order to make the model better understood, we used the Class Activation Mapping (CAM) method (Zhou et al., 2016) to visualize the important domains resulting in the decision of the model. After preprocessing, such as removing noise and rescale, the region heat maps were fully generated by a deep learning model without manual annotation. We selected a typical image from each of the three disease types to show in Figure 3. The first column displays the original image and heat map of normal control patients from top to bottom. The second column demonstrates the original image and heat map of other pneumonia patients and the third column shows images of COVID-19. The heat maps are standard jet color pictures made by OpenCV and are overlapped on the initial image, where dark red highlights the activation regions associated with the classification.

### Performance of Predicting Mild and Moderate Types of COVID-19

After establishing a model that can quickly and accurately diagnose COVID-19, we also want to further establish an automatic classification system so that a truly integrated diagnosis and treatment can be realized, helping doctors determine whether the patient is suffering from COVID-19 at the shortest time possible after receiving the patient. If the person is suffering from COVID-19, what kind of clinical type is it? The most suitable treatment can then be taken on time.

**FIGURE 4:**
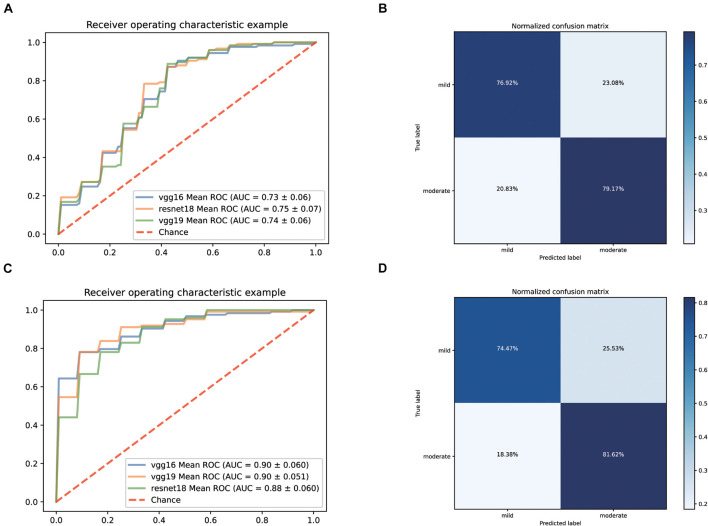
Performance of our AI Systems in predicting mild and moderate types of COVID-19 based on the original CT images and on the original CT images plus biochemical indicators. **(A,B)** ROC curves and normalized confusion matrix of one model (resnet18) based on the original CT images. **(C,D)** ROC curves and normalized confusion matrix (vgg19) based on the original CT images and biochemical indicators.

Firstly, we still predicted the clinical classification of patients (mild or moderate) based on the CT images on admission. Since the number of severe patients collected was too low, this type was excluded temporarily. After screening, we collected 495 qualified CT films from 61 mild patients and 470 qualified CT films from 125 moderate patients. The fivefold cross-validation method is still used to construct the classification system based on three pre-trained networks (vgg19, vgg16, and resnet18). The ROC curves of the classification systems are shown in Figure 4A. Among them, the model based on resnet18 has the highest AUC value (0.75). Its confusion matrix is shown in Figure 4B, with a sensitivity of 76.92% and a specificity of 79.17%. The AUC values of the other two models were 0.73 (vgg16) and 0.74 (vgg19), respectively. According to these results, it could be seen that the performance of the model constructed only based on CT images was not good enough for clinical application.

Considering that there was still a lot of routine examination information available on admission, we combined the information and CT images as input to establish a predictive classification model. The ROC curve of these three models we built is shown in Figure 4C. The performance of all models greatly improved after combining the laboratory findings, with AUC values ranging from 0.88 to 0.90. The confusion matrix of vgg19, which had the best AUC value, is displayed in Figure 4D, with a sensitivity of 74.47% and a specificity of 81.62% on the test set.

### Correlations of Lung Imaging Features and Laboratory Findings of COVID-19

**FIGURE 5:**
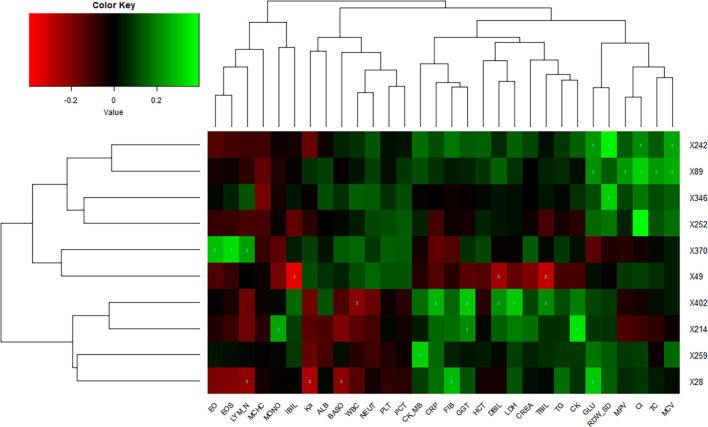
Correlations of lung imaging features and laboratory findings. Horizontal axis represents biochemical index, and vertical axis represents CT features. Number one indicates *p* < 0.001, number two indicates *p* < 0.01, and number three indicates *p* < 0.05.

Lung imaging features could reflect values of clinical biochemical parameters to some extent. Zhang et al. (2020) also reported that volume lesion ratio of lung was well linearly correlated to clinical parameters, such as C-reactive protein (CRP) and albumin. In order to investigate the association between CT features and biochemical indicators, we performed Spearman’s correlation tests and found key biochemical markers. The threshold of significant correlation was *p* < 0.05 after correction with the Holm–Bonferroni method, then the *p*-value was further refined into *p* < 0.001 (marked as 1), *p* < 0.01 (marked as 2), and *p* < 0.05 (marked as 3), which are labeled in Figure 5. In addition, we selected a |correlation value| of CT feature ≥0.35 and a |correlation value| of laboratory findings ≥0.30 to show in Figure 5. Eosinophil ratio (EO%), eosinophils (EOS), and lymphocyte number (LYM_N) showed highly positive correlations with the X370 feature of CT (lesion features). Generally speaking, the increase of eosinophils and lymphocyte number at the same time is considered to be caused by virus infection, which may be reflected on CT images of the lung. Glucose (GLU), red cell distribution width standard deviation (RDW_SD), chlorine (Cl), and mean corpuscular volume (MCV) were highly correlated with the X242 and X89 feature of CT. CRP, γ-glutamyl transpeptidase (GGT), direct bilirubin (DBIL), lactic dehydrogenase (LDH), and total bilirubin (TBIL) were highly correlated with the X402 feature of CT, whereas indirect bilirubin (IBIL), DBIL, and TBIL showed highly negative correlations with the X49 feature of CT.

This means the damage of liver and heart can be reflected in lung imaging as these biochemical indexes are markers of liver and heart function, suggesting that pulmonary lesions are not only related to the function of the respiratory system but also related to the health of other major organs, although we did not know the exact underlying pathogenetic mechanisms.

## Discussion

In this study, we collected CT images of all patients with COVID-19 in Inner Mongolia since the outbreak and CT images of other common pneumonia and normal controls who visited the hospital 6 months before the outbreak of COVID-19. Based on these CT images, we applied three network structures to construct a deep learning model for disease classification. Among these, vgg19 represented the best performance with an AUC of 0.95, which provided a favorable tool for rapid diagnosis of COVID-19. It was conceivable that if this model was applied to medical units, it would help radiologists and clinicians fight against the pandemic and reduce their burden. Especially in remote areas or communities where there was lack of experienced doctors, it would be an effective complementary measure. For patients with COVID-19, we further developed a deep learning model to predict the clinical classification through CT images of the first examination after admission. However, the classification effect of the prediction model based on three network structures was lower than expected, so the laboratory findings were added to improve the prediction effect and the performance of vgg19 was raised to an AUC of 0.90. This would be conducive to the timely arrangement of an appropriate treatment plan according to the patient’s condition to achieve the efficient management of patients. Thus, based on the routine examination items of patients, we built a rapid identification and classification system of COVID-19. In addition, we found that CRP, EOS, LDH, and other indicators were significantly correlated with the CT image features of the lung.

Our research still had some limitations. Firstly, the sample size used to build the model was relatively small. Deep learning typically needed a large number of samples to extract features and the training model in order to achieve wider applicability and higher accuracy. Our model put forward the feasibility of using CT images to predict the trend and classification of COVID-19 disease, and more samples are needed to optimize the model and test the generalizability of the an AI model. The cooperation with more medical centers or hospitals may contribute to the improvement of this work. Secondly, as the number of severe and critical patients was too few, their CT slices were not included in this study. The model still needed to be improved in predictable subtypes and disease severity. If we could collect more CT images of severe patients to train the model, this study will include more comprehensive types of prediction and will be more applicable in clinical practice. Thirdly, we adopted the chest CT slices directly without preprocessing of lung segmentation in order to save time, while lung segmentation preprocessing was generally regarded to improve the accuracy of AI training (Chung et al., 2018; Li et al., 2020; Xu et al., 2020). In weighing the advantages against the disadvantages, we chose transfer learning based on resnet18, vgg19, and vgg16 pre-trained CNN models in the ImageNet data sets (Shin et al., 2016) and fivefold cross-validation. Finally, besides early diagnosis, tracing the origin of SARS-CoV-2 is also critical for understanding and preventing the further outbreak of this virus (Li et al., 2021). There are many methods to identify the evolution of influenza that could be adopted in SARS-CoV-2 (Yang et al., 2013, 2014a). In addition, ideas like antigenic map (Barnett et al., 2012; Huang et al., 2017) and sequence-based virus antigenicity prediction (Sun et al., 2013; Yang et al., 2014b; Yao et al., 2017) are helpful for the vaccine design of SARS-CoV-2. More importantly, drug–target interaction identification (Peng et al., 2017; Zhou et al., 2019) based on the existing two targets (SARS-CoV-2 spike protein and human ACE2) may contribute to the prevention of COVID-19. However, it is out of the scope of this study.

After analyzing the correlation between clinical features and CT image features, we found that there were some features with high positive or negative correlation. As we did not extract and specify image features, such as GGO and consolidation, but chose the pixel feature of the image as the input, we could get a more comprehensive analysis. However, it was difficult to explain how the association was made. In future studies, we will collect more samples from hospitals or public databases to further optimize the performance of AI systems and extend the applicability.

## Data Availability Statement

The original contributions presented in the study are included in the article/supplementary material, further inquiries can be directed to the corresponding author.

## Ethics Statement

The studies involving human participants were reviewed and approved by the Medical Research Ethics Committee of Inner Mongolia People’s Hospital. Written informed consent from the participants’ legal guardian/next of kin was not required to participate in this study in accordance with the national legislation and the institutional requirements.

## Author Contributions

DS designed the study. LY, XL, WJ, XJ, and SX collected and analyzed the data. LY and XS interpreted the data and wrote the manuscript. AW, TL, XZ, and GT reviewed the manuscript. All authors contributed to the article and approved the submitted version.

## Conflict of Interest

XS, SX, AW, TL, and GT were employed by Geneis (Beijing) Co., Ltd. The remaining authors declare that the research was conducted in the absence of any commercial or financial relationships that could be construed as a potential conflict of interest.

## Publisher’s Note

All claims expressed in this article are solely those of the authors and do not necessarily represent those of their affiliated organizations, or those of the publisher, the editors and the reviewers. Any product that may be evaluated in this article, or claim that may be made by its manufacturer, is not guaranteed or endorsed by the publisher.
